# Effectiveness of Fluoride Varnish Versus Conventional Glass Ionomer in Preventing Occlusal Caries: A Systematic Review and Meta-Analysis

**DOI:** 10.7759/cureus.102436

**Published:** 2026-01-27

**Authors:** Abdullah Dh Alharbi, Masoud Almasoud, Fahad Alfadhli, Abdullah N Alharbi, Talal Aldhufairi, Rahaf Kh Alrashidi, Aisha Alameer, Abdulsalam Alenezi, Yousef Alqattan, Ahmed Abdelaziz

**Affiliations:** 1 Department of Dentistry, Taima Polyclinic, Ministry of Health, Al-Jahra, KWT; 2 Department of Dentistry, Abdulrahman Alzaid Polyclinic, Ministry of Health, Hawalli, KWT; 3 Department of Dentistry, Saad Al-Abdullah Polyclinic Block 10, Ministry of Health, Al-Jahra, KWT; 4 Department of Dentistry, Sabah Al-Salem Polyclinic, Mubarak Al Kabeer, KWT; 5 Department of Biostatistics, Faculty of Medicine, Al-Azhar University, Cairo, EGY

**Keywords:** fluoride varnish, glass ionomer cement, occlusal caries, permanent molars, visible plaque index

## Abstract

Fluoride varnish (FV) and conventional glass ionomer cement (GIC) sealants are widely used strategies for the prevention of occlusal caries in newly erupted permanent molars. However, their comparative effectiveness remains unclear. We aimed to evaluate the preventive efficacy of FV versus GIC sealants in pediatric populations. PubMed, Web of Science (WoS), Scopus, and Cochrane CENTRAL were systematically searched through January 2026 to identify randomized controlled trials (RCTs) comparing FV and GIC sealants. The primary outcome was the incidence of occlusal caries involving dentin ( International Caries Detection and Assessment System (ICDAS) ≥4). Secondary outcomes included caries development on adjacent second molars, plaque accumulation measured by the Visible Plaque Index (VPI), and patient-reported levels of anxiety and pain during application. Standardized mean differences (SMDs) and risk ratios (RRs) with 95% confidence intervals (CIs) were computed using random-effects models. Five RCTs including 1,626 patients and 5,060 teeth met the inclusion criteria. The incidence of occlusal caries and second molar caries, as well as anxiety and pain levels, was comparable between the FV and GIC groups. Patients receiving FV had modestly higher plaque scores compared to those receiving GIC (SMD = 0.11, 95% CI: 0.01 to 0.21, p = 0.03). Both FV and GIC sealants provide effective protection against occlusal caries in newly erupted permanent molars, with FV demonstrating a slight disadvantage in plaque accumulation. These findings suggest that clinicians can select either modality based on logistical considerations, patient preference, and resource availability, without compromising clinical effectiveness.

## Introduction and background

Dental caries is considered a prevalent chronic disease in pediatric populations worldwide and continues to represent a public health burden despite advances in preventive dentistry [1]. The surfaces of newly erupted permanent molars in children are particularly vulnerable to caries due to their complex pit-and-fissure morphology, prolonged eruption phase, and difficulty in maintaining adequate plaque control during early eruption [2]. These anatomical and behavioral factors make these teeth a primary target for preventive interventions [3].

Fluoride varnish (FV) and pit-and-fissure sealants are among the most widely recommended strategies for occlusal caries prevention [4]. FV exerts its protective effect through enhancement of enamel remineralization and inhibition of demineralization, whereas sealants provide a physical barrier that isolates pits and fissures from the oral environment. Conventional glass ionomer cement (GIC) sealants offer additional advantages over resin-based materials, including fluoride release, chemical adhesion to enamel, and tolerance to moisture contamination, making them particularly suitable for use in partially erupted molars and community-based settings [5].

Although both FV and glass ionomer sealants are extensively utilized in clinical practice and public health programs, uncertainty remains regarding their comparative effectiveness for occlusal caries prevention [6]. Individual randomized trials have reported inconsistent findings, and existing systematic reviews have primarily focused on resin-based sealants or combined multiple fluoride delivery methods, limiting the applicability of their conclusions to conventional glass ionomer sealants [6,7].

A clear synthesis of evidence specifically comparing FV and conventional glass ionomer sealants is therefore clinically important to inform preventive strategies, particularly in pediatric populations and resource-limited settings where material selection, cost-effectiveness, and ease of application are critical considerations.

Therefore, this systematic review and meta-analysis aimed to compare the impact of FV versus conventional glass ionomer sealants in preventing occlusal caries in permanent molars.

## Review

Methods and materials

We conducted this systematic review and meta-analysis according to the predefined criteria of the Preferred Reporting Items for Systematic Reviews and Meta-Analyses (PRISMA) [8] and the proposed methodologies in the Cochrane Handbook for Systematic Reviews of Interventions [9].

Literature Search and Screening

We conducted a systematic search of PubMed, Scopus, Web of Science (WoS), and Cochrane CENTRAL through January 2026 to identify randomized controlled trials (RCTs) using the following search terms: (glass ionomer sealant OR glass-ionomer sealant OR glass ionomer cement sealant OR GIS) AND (fluoride varnish OR sodium fluoride varnish OR NaFV OR Duraphat) AND (dental caries OR occlusal caries OR caries prevention OR caries progression OR ICDAS) AND (child OR preschool OR pediatric OR paediatric OR primary teeth OR primary molars). The specific search strategy for each database is presented in Table 1.

**Table 1:** Search strategy for each database.

Database	Keywords	Results
PubMed	(glass ionomer sealant OR glass-ionomer sealant OR glass ionomer cement sealant OR GIS) AND (fluoride varnish OR sodium fluoride varnish OR NaFV OR Duraphat) AND (dental caries OR occlusal caries OR caries prevention OR caries progression OR ICDAS) AND (child OR preschool OR pediatric OR paediatric OR primary teeth OR primary molars)	N = 35
Scopus	TITLE-ABS-KEY ( ( "glass ionomer sealant" OR "glass-ionomer sealant" OR "glass ionomer cement sealant" OR GIS ) AND ( "fluoride varnish" OR "sodium fluoride varnish" OR NaFV OR Duraphat ) AND ( "dental caries" OR "occlusal caries" OR "caries prevention" OR "caries progression" OR ICDAS ) AND ( child OR preschool OR pediatric OR paediatric OR "primary teeth" OR "primary molars" ) )	N = 18
Web of Science (WOS)	TS=(("glass ionomer sealant" OR "glass-ionomer sealant" OR "glass ionomer cement sealant" OR GIS) AND ("fluoride varnish" OR "sodium fluoride varnish" OR NaFV OR Duraphat) AND ("dental caries" OR "occlusal caries" OR "caries prevention" OR "caries progression" OR ICDAS) AND (child OR preschool OR pediatric OR paediatric OR "primary teeth" OR "primary molars")	N = 15
Cochrane CENTRAL	("glass ionomer sealant" OR "glass-ionomer sealant" OR GIS) AND ("fluoride varnish" OR "sodium fluoride varnish" OR NaFV) AND (child OR preschool OR pediatric)	N = 1

In addition, the reference lists of all retrieved articles and relevant reviews were manually screened to identify additional potentially eligible trials. Study selection was performed in two stages. First, duplicate records were removed using EndNote software (Clarivate Analytics, Philadelphia, Pennsylvania). Second, titles and abstracts were screened independently by two reviewers, followed by full-text assessment of potentially eligible articles. Any discrepancies were resolved through discussion or consultation with a third reviewer.

Eligibility Criteria

We included all studies meeting the following PICO criteria: (1) population: children and adolescents with newly erupted primary or permanent molars who were caries-free at baseline; (2) intervention: application of FV; (3) control: application of conventional GIC sealants; and (4) outcomes: the endpoints of interest. The studies had to be RCTs. We excluded non-randomized studies, observational designs, in vitro or animal studies, trials involving adults or teeth with existing dentinal caries, studies evaluating alternative preventive interventions (resin-based sealants or silver diamine fluoride), reports lacking relevant outcome data, studies with insufficient follow-up, and non-full-text publications such as conference abstracts, reviews, or editorials.

Endpoints

The primary endpoint of interest was the incidence of occlusal caries, defined as carious lesions progressing into dentin. Caries severity was assessed using the International Caries Detection and Assessment System (ICDAS), a validated visual scoring system ranging from 0 (sound surface; no evidence of decay) to 6 (extensive distinct cavity with visible dentin), based on visual examination after cleaning and drying of teeth [10], with scores ≥4 considered indicative of clinically significant dentin caries. Secondary outcomes included the development of caries on adjacent permanent molars, particularly second molars, and differences in plaque accumulation, measured by the Visible Plaque Index (VPI), a validated index assessing the presence of dental plaque on tooth surfaces [11]. In addition, patient-reported levels of anxiety and pain associated with the application of fluoride varnish or glass ionomer sealants, providing information on the acceptability and tolerability of these preventive strategies, were extracted when available.

Data Extraction

We used a standardized Excel sheet (Microsoft Corporation, Redmond, Washington) to extract all relevant data from the included studies. The extracted data were as follows: (1) summary characteristics of the included studies, including country, time frame, study design, inclusion and exclusion criteria, and key findings; (2) baseline characteristics of the patients included, such as sample size, age, sex, number of teeth included per patient, and frequency of tooth brushing, supervised tooth brushing, toothpaste use, snacking, night bottle habit, and previous dental visits; (3) risk of bias domains; and (4) studied endpoints.

Quality Assessment

Two reviewers assessed the quality of the included studies using the Cochrane risk of bias assessment tool version 2 (ROB-2) [12]. This tool incorporates five domains: bias arising from randomization, deviations from intended interventions, missing outcome data, measurement of the outcome, and selection of the reported results. Each domain was ranked as low risk, some concerns, or high risk of bias, with an overall risk ranking. Any discrepancies were resolved with the other author through discussion.

Statistical Analysis

Dichotomous outcomes were extracted as the number of events with total patients or teeth and were pooled as risk ratios (RRs) with 95% confidence intervals (CIs) using the DerSimonian-Laird random-effects model. For continuous outcomes, means and standard deviations (SDs), along with the total number of patients, were extracted. These data were pooled as standardized mean differences (SMDs) with 95% CIs using the same model. Significant heterogeneity was determined when the p-value was less than 0.05 and I2 ≥50%. STATA 19MP software (StataCorp LLC, College Station, Texas) was used to perform all statistical analyses.

The certainty of evidence for each outcome was assessed using the Grading of Recommendations Assessment, Development and Evaluation (GRADE) approach. Randomized controlled trials were initially rated as high-certainty evidence and subsequently downgraded based on predefined criteria, including risk of bias, inconsistency, indirectness, imprecision, and potential publication bias. Certainty ratings were categorized as high, moderate, low, or very low. Summary of Findings tables were generated to present the pooled effect estimates and corresponding certainty of evidence for all primary and secondary outcomes.

Results

Literature Review and Screening

**Figure 1:**
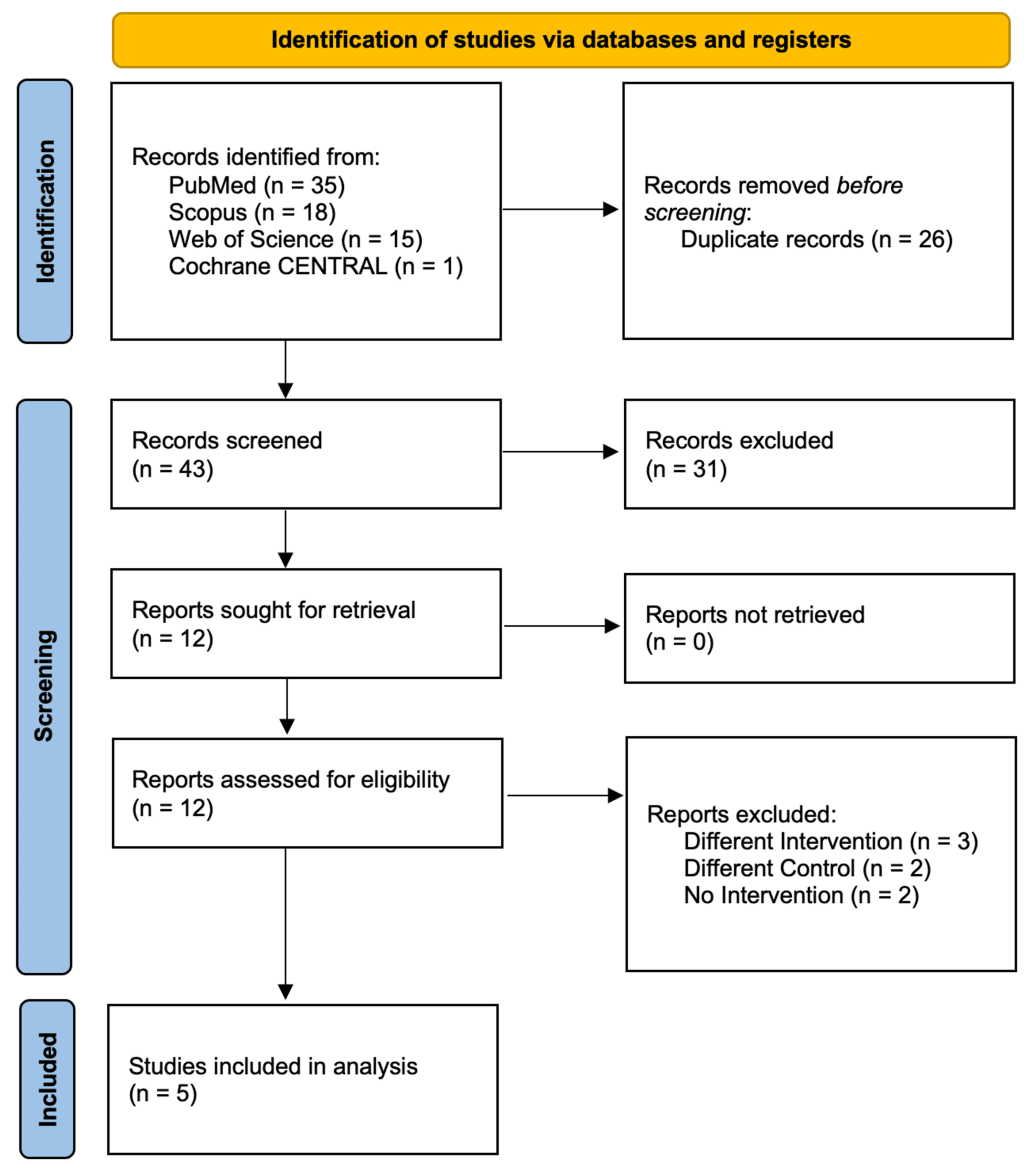
The PRISMA flow chart. PRISMA: Preferred Reporting Items for Systematic Reviews and Meta-Analyses.

Our comprehensive search across the databases yielded 69 citations, of which 57 were removed following title and abstract screening and duplicate removal, leaving 12 citations for full-text screening. Of these, five RCTs met the inclusion criteria and were included in the analysis [6,13-16]. The PRISMA flow chart is shown in Figure 1.

Characteristics and Quality Assessment

The five RCTs finally included were conducted in three countries: Hong Kong, India, and Brazil. A total of 1,626 patients were included, with a mean patient age of 4.5 years. Of these, 802 patients (49.32%) were treated with GIC sealants, including 2,230 teeth; 744 patients (45.76%) were treated with FV, including 2,508 teeth; and 80 patients (4.92%), including 320 teeth, were treated using a split-mouth design. Detailed baseline and summary characteristics of the included patients and studies are presented in Tables 2, 3.

**Table 2:** Summary characteristics of the included studies. PSM: primary second molar, ICDAS: International Caries Detection and Assessment System, RCT: randomized controlled trial, GIS: glass ionomer sealant, NaFV: sodium fluoride varnish, RMGI-F: resin-modified glass ionomer–fluoride.

Study	Country	Time frame	Study Design	Patients, n			Inclusion criteria	Exclusion criteria	Key findings
				Total	Control	Intervention			
Lam 2021 [13]	Hong Kong	12 months	RCT, parallel group	323	169	154	Aged 3–4 years, moderate-high caries risk, ≥1 PSM with ICDAS 0–3	Systemic disease, long-term medication, uncooperative, recent fluoride treatment, PSM with ICDAS ≥4	No significant difference in caries progression between GIS and NaFV (7.8% vs 8.0%). GIS retention: 24.6% (6m), 13.5% (12m).
Lam 2024 [6]	Hong Kong	18-24 months	RCT, parallel group	736	353	383	Aged 3–4 years, moderate-high caries risk, ≥1 sound PSM, cooperative	Special healthcare needs, long-term meds, uncooperative, PSM with ICDAS ≥4 or restored	No significant difference in caries prevention between GIS and NaFV (17.1% vs 17.0% dentinal caries). GIS retention dropped to 2.1% at 18–24m.
Chiu 2025 [14]	Hong Kong	Single visit	RCT, parallel group	413	185	228	Aged 3–6 years, moderate-high caries risk, cooperative	Systemic disease, special needs, uncooperative, recent fluoride treatment	NaFV associated with less anxiety and better cooperation than GIS. Similar pain levels. NaFV faster to apply.
Uchil 2021 [16]	India	12 months	RCT, parallel group	74	37	37	Aged 6–8 years, partially erupted permanent first molars with ICDAS 0–3, high caries risk, cooperative	Medical conditions affecting saliva, orthodontic treatment, allergies to resins	No significant difference in caries prevention (ICDAS ≥1) between RMGI-F varnish and GIS. Retention similar.
Oliveira 2013 [15]	Brazil	18 months	RCT, split mouth	80	80	80	Aged 6–8 years, all four newly erupted sound permanent first molars	Not specified beyond caries experience grouping	Caries incidence similar between GIS and FV (15 vs 13 teeth). GIS retention very low (1% full, 69% partial at 18m).

**Table 3:** Baseline characteristics of the included patients. NA: not assigned, dft score: decayed and filled teeth score, dt score: decayed teeth score, ft score: filled teeth score.

Group	Category	Lam 2021 [13]		Lam 2024 [6]		﻿Chiu 2025 [14]		Oliveira 2013 [15]		Uchil 2021 [16]	
		Control	Intervention	Control	Intervention	Control	Intervention	Control	Intervention	Control	Intervention
Sample Size, n		169	154	353	383	185	228	80		37	37
Age, months, mean (SD)		46.3 (3.7)	46.5 (3.7)	48.9 (7.2)	49.4 (7.2)	51.3 (8.6)	51.4 (8.2)	6-8 years		81.6 (9.6)	80.4 (8.4)
Sex, n (%)	Male	96 (56.8%)	76 (49.4%)	184 (52.1%)	198 (51.7%)	89 (48.1%)	122 (53.5%)	NA	NA	17 (45.9%)	19 (51.4%)
	Female	73 (43.2%)	78 (50.6%)	169 (47.9%)	185 (48.3%)	96 (51.9%)	106 (46.5%)	NA	NA	17 (45.9%)	19 (51.4%)
﻿Grade at baseline, n (%)	﻿Kindergarten grade 1	61 (﻿36.1)	48 (﻿31.2%)	227 (64.3%)	240 (62.7%)	59 (31.9%)	85 (37.3%)	NA	NA	NA	NA
	﻿Kindergarten grade 2	86 (﻿50.9)	87 (﻿56.5%)	126 (35.7%)	143 (37.3%)	126 (68.1%)	143 (62.7%)	NA	NA	NA	NA
Number of teeth included per patient, n		514	475	813	951	665	845	320		87	89
﻿Tooth brushing ﻿frequency, n (%)	﻿Less than once a day	13 (7.7%)	15 (9.7%)	22 (6.2%)	28 (7.3%)	9 (4.9%)	13 (5.7%)	NA	NA	NA	NA
	﻿Once a day	55 (32.5%)	50 (32.5%)	100 (28.3%)	122 (31.9%)	45 (24.3%)	72 (31.6%)	NA	NA	16 (43.2%)	23 (62.2%)
	﻿Twice or more a day	96 (59.2%)	89 (57.8%)	222 (62.9%)	217 (56.7%)	127 (68.6%)	136 (59.6%)	NA	NA	21 (56.8%)	14 (37.8%)
﻿Supervised toothbrush, n (%)	﻿No	13 (7.7%)	15 (9.7%)	29 (8.2%)	31 (8.1%)	17 (9.2%)	16 (7.0%)	NA	NA	NA	NA
	﻿Yes, sometimes	77 (45.6%)	56 (36.4%)	145 (41.1%)	138 (36.0%)	68 (36.8%)	81 (35.5%)	NA	NA	NA	NA
	﻿Yes, all the time	78 (46.2%)	81 (52.6%)	176 (49.9%)	210 (54.8%)	98 (53.0%)	129 (56.6%)	NA	NA	NA	NA
﻿Toothpaste, n (5)	﻿No toothpaste	10 (5.9%)	14 (9.1%)	151 (42.8%)	178 (46.5%)	9 (4.9%)	16 (7.0%)	NA	NA	NA	NA
	﻿Child nonfluoridated toothpaste	55 (32.5%)	56 (36.4%)	140 (39.7%)	154 (40.2%)	77 (41.6%)	92 (40.4%)	NA	NA	NA	NA
	﻿Child or adult fluoridated toothpaste	72 (42.6%)	64 (41.6%)	7 (2%)	3 (0.8%)	76 (41.1%)	92 (40.4%)	NA	NA	26 (70.3%)	27 (73.0%)
	﻿Uncertain	30 (17.8%)	17 (11.0%)	55 (15.6)	48 (12.5)	23 (12.7)	28 (12)	NA	NA	11 (29.7%)	10 (27.0%)
﻿Snacking frequency, n (%)	﻿Less than once a day	19 (12.4%)	17 (11.0%)	36 (10.2%)	43 (11.2%)	15 (8.1%)	26 (11.4%)	NA	NA	NA	NA
	﻿Once a day	40 (23.7%)	52 (33.8%)	93 (26.4%)	124 (32.4%)	53 (28.6%)	72 (31.6%)	NA	NA	NA	NA
	﻿Twice a day	72 (42.6%)	54 (35.1%)	156 (44.2%)	156 (40.7%)	84 (45.4%)	101 (44.3%)	NA	NA	NA	NA
	﻿Over twice	35 (20.7%)	31 (20.1%)	68 (19.3%)	60 (15.7%)	33 (17.8%)	29 (12.7%)	NA	NA	NA	NA
﻿Night bottle habit, n (%)	﻿Yes	19 (11.2%)	17 (11.0%)	34 (9.6%)	43 (11.2%)	6 (3.2%)	11 (4.8%)	NA	NA	NA	NA
	﻿Previously yes but winded up recently	51 (30.2%)	46 (29.9%)	92 (26.1%)	95 (24.8%)	10 (5.4%)	15 (6.6%)	NA	NA	NA	NA
	﻿Never	96 (56.8%)	90 (58.4%)	223 (63.2%)	241 (62.9%)	127 (68.6%)	150 (65.8%)	NA	NA	NA	NA
﻿Previous dental visit, n (%)	No	149 (88.2%)	129 (83.8%)	300 (85.0%)	321 (83.8%)	152 (82.2%)	191 (83.8%)	NA	NA	31 (83.8%)	30 (81.1%)
	Yes	20 (11.8%)	25 (16.2%)	51 (14.4%)	61 (15.9%)	31 (16.8%)	36 (15.8%)	NA	NA	6 (16.2%)	7 (18.9%)
﻿dft score, mean (SD)		1.2 (2.5)	1.2 (2.7)	1.3 (2.6)	1.4 (2.6)	1.4 (2.8)	1.5 (2.6)	NA	NA	NA	NA
﻿dt score, mean (SD)		1.2 (2.5)	1.2 (2.7)	1.3 (2.5)	1.3 (2.6)	1.4 (2.6)	1.5 (2.6)	NA	NA	NA	NA
﻿ft score, mean (SD)		0.0 (0.1)	0.0 (0.1)	0.0 (0.3)	0.0 (0.2)	0.0 (0.1)	0.0 (0.2)	NA	NA	NA	NA

**Figure 2:**
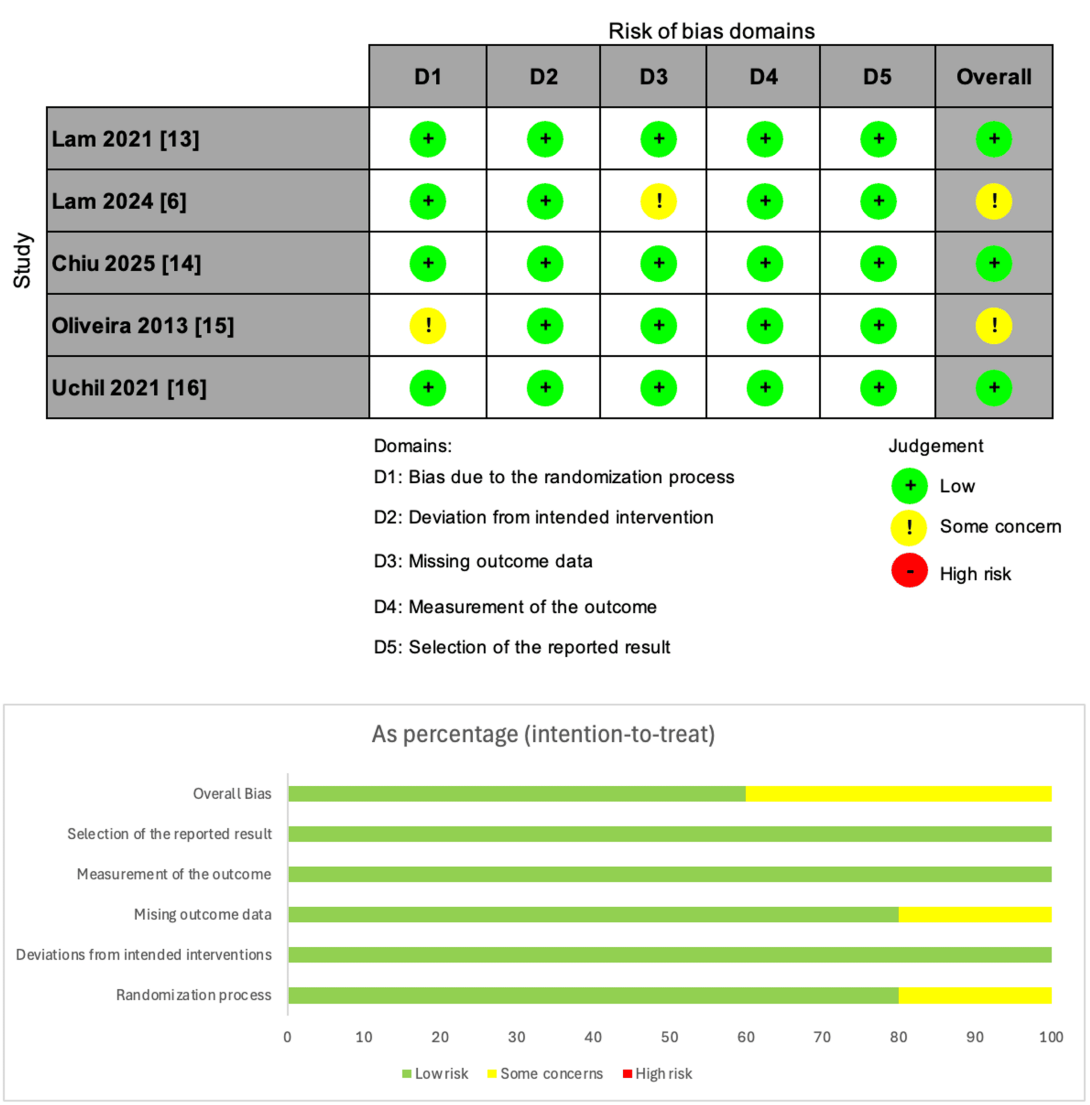
Risk of bias assessment using ROB-2 tool.

All RCTs were assessed for quality and risk of bias using ROB-2, as shown in Figure 2. Three studies had an overall low risk of bias, and the other two had overall some concerns of bias due to missing outcome data in one study and bias arising from the randomization process in the other.

Outcomes

**Figure 3:**
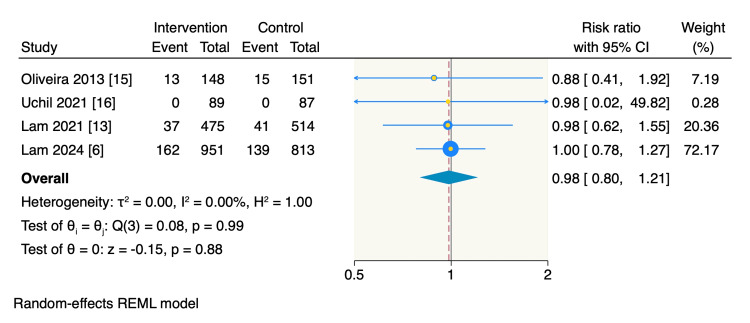
Random-effect model meta-analysis of the incidence of the occlusal caries. The data of the total refers to the teeth number.

Occlusal caries. Four RCTs reported the incidence of occlusal caries, which was similar between the intervention group (12.7%, 212 of 1663 teeth) and the control group (12.4%, 195 of 1565 teeth). The pooled estimate showed no significant difference between the intervention and control groups regarding occlusal caries (RR = 0.98, 95% CI: 0.80 to 1.21, p = 0.88; I^2^ = 0%), as shown in Figure 3.

**Figure 4:**
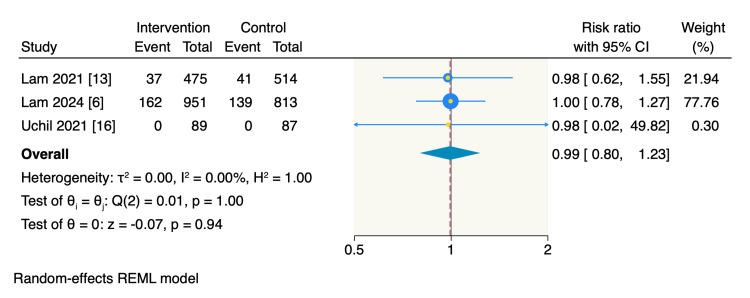
Random-effect model meta-analysis of the incidence of second molar caries. The data of the total refers to the teeth number.

Caries of second molars. Only three RCTs assessed the event rate of second molar caries, with similar rates observed between the intervention and control groups, at 13.1% (199 of 1515 teeth) and 12.7% (180 of 1414 teeth), respectively. The pooled estimate showed no significant difference between the two groups (RR = 0.99, 95% CI: 0.80 to 1.23, p = 0.94; I^2^ = 0%), as shown in Figure 4.

**Figure 5:**
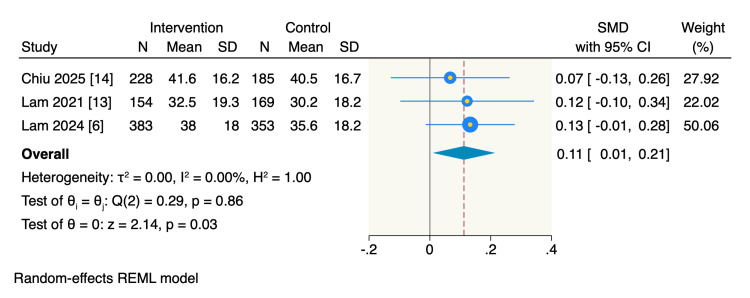
Random-effect model meta-analysis of differences in plaque accumulation, measured by the Visible Plaque Index (VPI). The data of the total refers to the patients number.

Visible Plaque Index. Three RCTs reported differences in plaque accumulation measured by VPI. The pooled estimate showed a significant increase in VPI scores among patients in the intervention group compared with those in the control group (SMD = 0.11, 95% CI: 0.01 to 0.21, p = 0.03; I^2^ = 0%), as shown in Figure 5.

**Figure 6:**
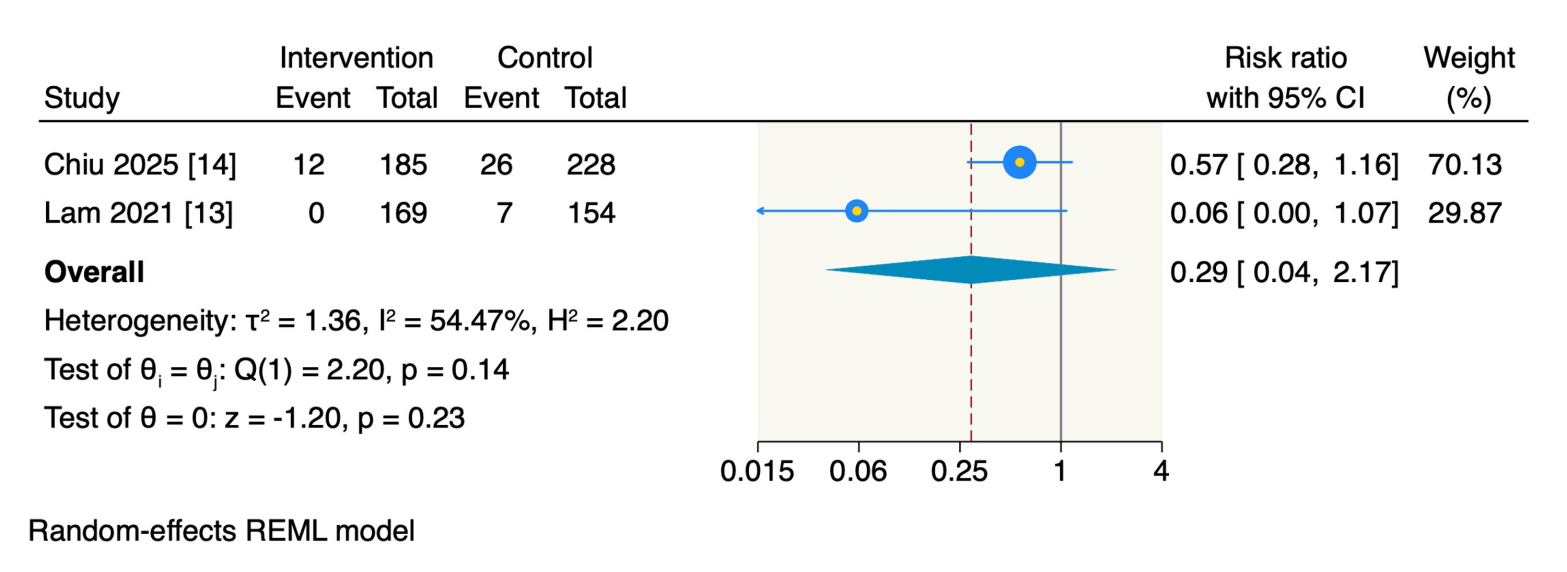
Random-effect model meta-analysis of the incidence of anxiety and pain levels of the patients. The data of the total refers to the patients number.

Anxiety and pain. Two RCTs assessed patient anxiety and pain levels. The pooled estimate showed no significant difference between the intervention and control groups (RR = 0.29, 95% CI: 0.04 to 2.17, p = 0.23; I^2^ = 54.47%), as shown in Figure 6.

GRADE Assessment

Using the GRADE framework, the certainty of evidence was rated as moderate for occlusal caries incidence and the VPI, primarily due to imprecision related to confidence intervals and the limited number of contributing studies. The certainty of evidence for caries development in second molars and patient-reported anxiety and pain was rated as low, reflecting additional concerns regarding indirectness, inconsistency, and serious imprecision. Overall, while randomized evidence was available for all outcomes, methodological and statistical limitations reduced confidence in the precision of effect estimates, particularly for secondary clinical and patient-reported outcomes (Table 4).

**Table 4:** GRADE assessment. GRADE: Grading of Recommendations Assessment, Development and Evaluation.

Outcome	No. of studies (RCTs)	No. of participants /teeth	Relative effect (95% CI)	Absolute effect	Certainty of evidence (GRADE)	Reasons for downgrading
Occlusal caries incidence	4	3,228 teeth	RR 0.98 (0.80–1.21)	2 fewer per 1,000 (from 25 fewer to 26 more)	Moderate ⨁⨁⨁◯	Imprecision (CI includes benefit and harm)
Second molar caries	3	2,929 teeth	RR 0.99 (0.80–1.23)	1 fewer per 1,000 (from 25 fewer to 29 more)	Low ⨁⨁◯◯	Imprecision; indirectness
Visible Plaque Index (VPI)	3	—	SMD 0.11 (0.01–0.21)	Small increase in plaque accumulation	Moderate ⨁⨁⨁◯	Imprecision (small effect size, limited studies)
Anxiety and pain	2	—	RR 0.29 (0.04–2.17)	Uncertain effect	Low ⨁⨁◯◯	Serious imprecision; inconsistency

Discussion

The present systematic review and meta-analysis of RCTs compared the effectiveness of FV versus conventional GIC sealants in preventing occlusal caries in permanent molars. While both preventive modalities are widely used in clinical practice and public health programs, head-to-head evidence specifically contrasting FV and GIC sealants has been limited and inconclusive. Our findings suggest that, overall, there are no significant differences between FV and GIC sealants in most clinically relevant outcomes, including the incidence of occlusal caries, second molar caries, and patient-reported levels of anxiety and pain. In addition, plaque accumulation, measured by VPI, was higher in the FV group.

Our analysis suggests that fluoride varnish and conventional glass ionomer sealants provide comparable protection against the development of clinically significant occlusal caries in newly erupted permanent molars. This finding aligns with several individual trials that have reported similar preventive efficacy for FV and GIC sealants when used in high-risk pediatric populations [6,14]. One possible explanation for this lack of difference is that both interventions mitigate the primary etiological factors of caries, plaque colonization, and acid demineralization through distinct mechanisms. Fluoride varnish primarily promotes remineralization and increases enamel resistance to acid, whereas GIC sealants provide a physical barrier to protect fissures and also release fluoride over time [17,18]. When the balance of demineralization and remineralization is maintained through periodic reapplication of FV or regular monitoring of GIC sealant retention, both approaches may effectively prevent progression of lesion severity [19,20]. In addition, many of the included studies supplemented FV and GIC with additional preventive measures, such as oral hygiene instructions and professional fluoride applications, which may have minimized detectable differences between groups.

The analysis of caries development on adjacent second molars also revealed comparable outcomes between fluoride varnish and conventional glass ionomer sealants. This suggests that the protective effects observed on first permanent molars may extend to adjacent erupting teeth regardless of the specific preventive modality [21]. It is important to note that second molar caries may be influenced by factors beyond the localized effect of fissure sealants or varnish, such as overall oral hygiene habits and dietary sugar intake, which may further dilute the impact of a single intervention [22].

Patient-reported levels of anxiety and pain associated with application of the two modalities were likewise comparable. While dental anxiety and discomfort can be barriers to preventive care, particularly in pediatric populations, neither FV nor GIC sealants appear to confer an advantage in terms of tolerability [23]. This result is consistent with prior evidence indicating that both FV application and sealant placement are generally well tolerated, with minimal discomfort during application [14]. From a practical standpoint, this suggests that patient preference or behavioral considerations may not need to heavily favor one modality over the other based solely on immediate procedural experience.

Analysis of plaque accumulation, as measured by VPI, indicated a modest but consistent increase in patients receiving fluoride varnish compared with those receiving conventional glass ionomer sealants. This may reflect differences in plaque ecology associated with repeated fluoride exposure [13]. Fluoride varnish has been shown to suppress cariogenic bacterial activity and alter plaque acidogenicity, potentially leading to changes in plaque accumulation over time [14]. Conversely, while GIC sealants release fluoride, their primary benefit is mechanical occlusion of fissures rather than modulation of plaque biofilm [24]. This increase in VPI in the FV group may indicate that GIC sealants have broader effects on the oral environment that extend beyond fissure sites.

The non-significant differences observed for most clinical outcomes may also be partially explained by methodological factors across studies. Many RCTs included in this meta-analysis had relatively short follow-up periods (≤12 to 18 months), which may not be sufficient to detect meaningful divergences in caries incidence that accrue over longer durations. Furthermore, variations in study design, patient age, baseline caries risk, sealant retention, frequency of FV reapplication, and adjunctive preventive measures could attenuate differences between FV and GIC. Importantly, several studies reported high rates of sealant loss over time, a known limitation of GIC sealants, yet caries prevention persisted. This apparent paradox reflects the caries-preventive benefit of fluoride release and remineralization, even when mechanical retention is imperfect [16].

Our study has several clinical implications. First, both FV and GIC sealants appear to be viable options for occlusal caries prevention in newly erupted permanent molars, especially in pediatric and community settings where moisture control may be challenging. Second, FV may offer an additional benefit in influencing plaque accumulation, which could contribute to broader oral health outcomes. Finally, the choice between FV and GIC sealants may be guided more by logistical considerations such as cost, ease of placement, and required expertise rather than clear differences in clinical effectiveness.

Limitations

Our study has several limitations. First, the number of RCTs reporting certain outcomes, such as anxiety, pain, and second molar caries, was small, which limits statistical power and precision. Second, the included studies were conducted in specific geographic and clinical settings, which may limit generalizability to broader or higher-risk populations. Third, differences in operator experience, assessment methods, and study protocols could have introduced notable measurement bias, particularly for subjective outcomes like VPI or patient-reported anxiety and pain. Finally, while validated, indices such as ICDAS and VPI are inherently operator-dependent and may be affected by examiner calibration or inter-rater variability, which could influence outcome consistency across trials.

## Conclusions

This systematic review and meta‑analysis of five RCTs of 1,626 patients suggests that fluoride varnish and conventional glass ionomer sealants demonstrate similar effectiveness in preventing occlusal caries and related clinical outcomes, with a modest disadvantage for FV in increasing plaque accumulation. These results support the continued use of either modality in clinical practice, tailored to patient needs and resource availability.
